# Prevention of neointimal formation using miRNA-126-containing nanoparticle-conjugated stents in a rabbit model

**DOI:** 10.1371/journal.pone.0172798

**Published:** 2017-03-02

**Authors:** Masayasu Izuhara, Yasuhide Kuwabara, Naritatsu Saito, Erika Yamamoto, Daihiko Hakuno, Yasuhiro Nakashima, Takahiro Horie, Osamu Baba, Masataka Nishiga, Tetsushi Nakao, Tomohiro Nishino, Fumiko Nakazeki, Yuya Ide, Masahiro Kimura, Takeshi Kimura, Koh Ono

**Affiliations:** Department of Cardiovascular Medicine, Graduate School of Medicine, Kyoto University, Kyoto, Japan; Qatar University College of Health Sciences, QATAR

## Abstract

**Background:**

Despite recent progress with drug-eluting stents, restenosis and thrombosis after endovascular intervention are still major limitations in the treatment of cardiovascular diseases. These problems are possibly caused by inappropriate inhibition of neointimal formation and retardation of re-endothelialization on the surface of the stents. miR-126 has been shown to have the potential to enhance vascular endothelial cell proliferation.

**Methods and results:**

We designed and constructed a 27-nt double strand RNA (dsRNA) conjugated to cholesterol, which has high membrane permeability, and formed mature miR-126 after transfection. For site-specific induction of miR-126, we utilized poly (DL-lactide-co-glycolide) nanoparticles (NPs). miR-126-dsRNA-containing NPs (miR-126 NPs) significantly reduced the protein expression of a previously identified miR-126 target, SPRED1, in human umbilical vascular endothelial cells (HUVECs), and miR-126 NPs enhanced the proliferation and migration of HUVECs. On the other hand, miR-126 NPs reduced the proliferation and migration of vascular smooth muscle cells, via the suppression of IRS-1. Finally, we developed a stent system that eluted miR-126. This delivery system exhibited significant inhibition of neointimal formation in a rabbit model of restenosis.

**Conclusions:**

miR-126 NP-conjugated stents significantly inhibited the development of neointimal hyperplasia in rabbits. The present study may indicate the possibility of a novel therapeutic option to prevent restenosis after angioplasty.

## Introduction

Currently, percutaneous coronary intervention is a major therapeutic option to treat ischemic heart diseases with low invasiveness. The introduction of drug-eluting stents (DESs) has effectively reduced in-stent restenosis (ISR). However, the rate of ISR has been reported as between 3% and 20%, depending on which DES is evaluated, the duration of follow-up, and the complexity of the lesions in which the stents were placed [1]. ISR usually presents as recurrent angina, but can present as acute myocardial infarction as well. Thus, patients with ISR require additional revascularization. In addition, DESs have been also implicated as having a risk of stent thrombosis (ST) associated with impaired vascular healing [2]. In clinical trial populations, the risk has been estimated at 0.2–0.3% per year after the first year [3], but a number of studies have demonstrated the risk beyond 1 year is higher in routine practice settings [4].

After angioplasty, several cellular and molecular events occur sequentially in the vascular wall [5]. The lack of endothelial cells disrupt the homeostatic regulation of thrombosis and leukocyte adhesion. Activation of vascular smooth muscle cells (VSMCs) is also caused by vascular damage, resulting in VSMC growth and extracellular matrix deposition, leading to the progression of neointimal hyperplasia [6]. Although several approaches have been proposed to regulate neointimal growth, modulation of a single molecule or pathway might only have limited success against this multifactorial process.

MicroRNAs (miRNAs; miRs) are small, single-stranded non-coding RNAs that act as post-transcriptional regulators of gene expression [7,8,9]. Each miRNA has been shown to regulate the expression of multiple genes. miR-126 is an endothelial cell-enriched abundant miRNA, and its expression is induced by the mechano-sensitive zinc finger transcription factor KLF2a. miR-126 is known to have the potential to promote the proliferation of endothelial cells by targeting SPRED1, an intracellular inhibitor of vascular endothelial growth factor signaling [10,11]. miR-126 also has anti-inflammatory effects on endothelial cells, because transfection of endothelial cells with an oligonucleotide that decreases miR-126 permitted an increase in tumor necrosis factor (TNF)-α-stimulated vascular adhesion molecule 1 (VCAM-1) expression and increased leukocyte adherence to endothelial cells [12]. We also found that miR-126 can inhibit VSMC proliferation and migration via the regulation of IRS-1.

From these results, we speculated that miR-126 induction is beneficial for inhibiting vascular injury, and we tried to examine the effect by using miR-126 double-stranded RNA (dsRNA) containing poly (DL-lactide-co-glycolide) (PLGA) nanoparticles (NPs) *in vitro*. Then, we further evaluated the effect of miR-126 dsRNA-containing NP (miR-126 NP)-conjugated stents in a rabbit model.

## Materials and methods

### Animal preparation

This investigation conformed to the Guide for the Care and Use of Laboratory Animals published by the US National Institutes of Health (NIH Publication No. 85–23, revised 1996). All animal care, experiments, and methods were approved by the Animal Care and Use Committees of Kyoto University Graduate School of Medicine.

### Cell culture

Primary cultures of human umbilical vein endothelial cells (HUVECs) were purchased from Kurabo and grown in Endothelial Cell Growth Medium 2 Kit (PromoCell, Heidelberg, Germany). Cells between passages 4 and 8 were used in experiments. HEK293 cells were cultured in Dulbecco’s modified Eagle’s medium (DMEM) supplemented with 10% FBS.

### Preparation of lipid-conjugated double-stranded RNAs

Recently, 27-nt dsRNAs have been found to exhibit much stronger gene-silencing effects than 21-nt siRNAs [15]. These 27-nt dsRNAs are cleaved by Dicer enzyme, leading to the release of 21-nt siRNAs, and thus are incorporated into the RISC. In order to overexpress miR-126, we adopted this method to induce dsRNAs to form mature miR-126. Moreover, we prepared cholesterol-conjugated 27-nt dsRNAs with the aim of facilitating cellular uptake and designed to stabilize mature miR-126 by phosphorothioate backbone modification. The following RNA oligonucleotides containing a partial phosphorothioate backbone (*) and a cholesterol group (Chol) were synthesized: miR-126 dsRNA sense, 5’Chol-CGGCGCAUUAUUACUCACGGUACGAGU-3’ and miR-126 dsRNA antisense, 5’ ACU(*)C(*)G(*)U(*)A(*)C(*)C(*)G(*)U(*)G(*)A(*)G(*)U(*)A(*)A(*)U(*)A(*)A(*)U(*)G(*)C(*)G(*)CCG-3’. The following control dsRNA contained a random sequence of nucleotides and was not complementary to any known miRNA: control dsRNA sense, 5’ Chol-CGGGUCUCCACGCGCAGUACAUUUCGU-3’ and control dsRNA antisense, 5’- ACG(*)A(*)A(*)A(*)U(*)G(*)U(*)A(*)C(*)U(*)G(*)C(*)G(*)C(*)G(*)U(*)G(*)G(*)A(*)G(*)A(*)C(*)CCG -3’. These dsRNAs were obtained from Hokkaido System Science (Japan).

### Preparation of PLGA NPs

The application of nanotechnology-based drug delivery systems (DDSs) is expected to have a major impact on the development of efficient and safe DDSs. It was reported that such a DDS was developed using a polymeric NP formulated from biodegradable PLGA, which can entrap hydrophilic agents such as oligonucleotides, penetrate cellular membranes via endocytosis, and deliver encapsulated therapeutic agents into the cellular cytoplasm. Biocompatible PLGA NPs were prepared using an emulsion solvent diffusion method, and the particle surface was modified with chitosan, as described previously [13,14]. Using this preparation method, dsRNA could be loaded on both the inner and outer layer of chitosan-modified PLGA NPs.

### Transfection

HUVECs were plated in a 6-well plate at 2.0×10^5^ cells per well. The cells were transfected with 2, 10, or 40 nM miR-126 dsRNAs or control dsRNAs using Lipofectamine 2000 (Invitrogen) for 48 h.

### Dual luciferase reporter assay

Three complementary tandem sequences for miR-126 were cloned into a pMIR-REPORT vector (Ambion). HEK293 cells were plated into 24-well plates and cotransfected with 0.4 μg of miR-126 luciferase reporter vectors and 2, 20, or 200 nM of miR-126 dsRNAs or 2 nM of control dsRNAs using Lipofectamine 2000. At 48 h after transfection, luciferase activity was measured using a dual luciferase reporter assay system (Toyo Ink). Firefly luciferase activity was normalized to Renilla luciferase activity. For the 3’-UTR assay, IRS-1 3’-UTR (WT) was inserted downstream of the firefly luciferase cassette in a pMIR-REPORT vector (Ambion). Mutated 3’-UTR (Mut) constructs were created using a QuikChange II Site-Directed Mutagenesis Kit (Agilent Technologies) in accordance with the manufacturer’s protocol. The miR-126 target site in the IRS-1 3’-UTR was mutated from GGTACGA to GCAAGCA. HEK293 cells were used for transfection. pMIR-REPORT vectors containing the WT or Mut IRS-1 3’-UTR were co-transfected with miR-126 or the negative control using TransIT-LT1 Transfection Reagent (Mirus Bio LLC).

### Isolation and culture of rabbit aortic smooth muscle cells

Rabbit aortic smooth muscle cells (RAoSMCs) were isolated from the abdominal aortas of Japanese white rabbits. Briefly, after administering anesthesia, the aorta was dissected from its origin and transferred into phosphate-buffered saline. The aorta was freed from connective tissue and adventitia and cut open longitudinally. The aorta was transferred to an empty collagen-coated dish and pressed with the luminal side down onto the plate. After complete adhesion to the plate was achieved, fresh medium was added slowly to the periphery of the plate until it just covered the aorta. The dish was then placed in a 37°C, in a 5% CO_2_ incubator and left undisturbed for 5 days. Cells were subcultured as needed for experiments. RAoVSMCs were maintained at 37°C with 5% CO_2_ in DMEM with High Glucose culture medium supplemented with 10% fetal bovine serum and 100 U/ml penicillin. Experiments were performed using RAoSMCs between passages 4 and 9.

### Intracellular uptake of NPs

The HUVECs and RAoSMCs were seeded onto a chamber slide to an initial concentration of 5.0 × 10^5^ cells per well and incubated at 37°C in a 5% CO_2_ environment. The growth medium was replaced with the FITC-NP suspension medium (0.5 mg/ml) and then further incubated for 3 hours. The cells were then washed 3 times with PBS to eliminate extracellular NPs. Then, the cells were fixed with 10% formaldehyde and nuclei were stained with 4’,6-diamidino-2-phenylindole. Intracellular uptake of FITC-NPs was evaluated by fluorescence microscopy (KEYENCE).

### Quantitative real-time PCR (qPCR) for miR and mRNA expression levels

Total RNA was isolated using Trizol (Invitrogen). miRNAs were quantified using TaqMan MicroRNA Assays (Applied Biosystems) and a StepOnePlus Real-Time PCR System (Applied Biosystems) in accordance with the manufacturer’s instructions. miRNA levels were normalized using U6 small nuclear RNA. For mRNA quantification, complementary DNA was synthesized using a Verso cDNA Synthesis Kit (Thermo Fisher Scientific) in accordance with the manufacturer’s instructions, and analysis of mRNA levels was performed on a StepOnePlus Real-Time PCR System (Applied Biosystems) with THUNDERBIRD SYBR qPCR Mix (TOYOBO). β-actin or GAPDH was used as a housekeeping gene. The primer sequences are listed in Online Table I.

### Western blotting analysis

Immunoblotting analysis was performed using standard procedures as described previously. Cells were lysed in lysis buffer [100 mM Tris-HCl (pH 7.4), 75 mM NaCl, and 1% Triton^™^ X-100 (Nacalai Tesque)] containing Complete Mini^™^ protease inhibitor (Roche), aprotinin (Sigma), 50 mM NaF, and 1 mM Na_3_VO_4_. Protein concentrations were determined using a bicinchoninic acid protein assay kit (Bio-Rad). For western blotting, 20 μg of protein was electrophoresed using NuPAGE^™^ 4–12% Bis-Tris (Invitrogen) gels and transferred to a Protran^™^ nitrocellulose transfer membrane (Whatman). The membrane was incubated with the primary antibody overnight at 4°C followed by incubation with the secondary antibody conjugated to horseradish peroxidase at a 1:2000 dilution. The signals were visualized using ECL-Plus^™^ chemiluminescent detection reagent (Amersham Biosciences). The following primary antibodies were used: anti-glyceraldehyde-3-phosphate dehydrogenase (GAPDH) (Cell Signaling, 14C10), 1:3000; anti-β-actin antibody (Sigma-Aldrich, AC-15), 1:3000; anti-SPRED1 antibody (abcam, M23-P2G3), 10 μg/ml; and anti-IRS-1 antibody (Cell Signaling, #2382), 1:1000. Anti-rabbit IgG (GE Healthcare) and anti-mouse IgG (GE Healthcare) were used as secondary antibodies, each at a dilution of 1:2000. Luminescence was detected using an ImageQuant LAS 4000mini (GE Healthcare). The densities of the band on western blots were quantified by using ImageJ software (NIH).

### Cell proliferation assay

HUVECs and RAoVSMCs proliferation *in vitro* was determined by cell counting and MTT assays. For cell counting, cells were plated in 6-well culture plates at 2.0×10^5^ cells per well. Either miR-126 NPs or control RNA NPs were added to medium at a concentration of 100 nM, and cells were incubated for 48 h. The cells were detached by trypsinization and resuspended in PBS. The cells were then counted using a Z1^™^ COULTER COUNTER (Beckman Coulter). For MTT assays, cells were seeded at a density of 1000 cells per well in 96-well culture plates for 24 h. miR-126 NPs or control RNA NPs were added to medium at a concentration of 100 nM. Cell proliferation was measured at intervals of 2 days. At the end of each time point, 10 μl of 5 mg/ml MTT (Dojindo Molecular Technologies) was added to each well. Four hours later, 100 μl acidic isopropanol (0.04 N HCl) was added to the MTT-treated wells and the absorption was determined at 595 nm (reference wavelength 690 nm) using an automated plate reader.

### Migration assay

HUVECs or RAoVSMCs were plated in a collagen type I-coated 35 mm glass-based dish (IWAKI) at a density of 1.0×10^5^ cells and either miR-126 NPs or control RNA NPs were added to medium at a concentration of 100 nM, and cells were incubated until a complete monolayer was formed. After serum starvation for 24 h, straight scratches for each dish were made using a 200 μl pipette tip. Cellular debris was removed by gently washing cells with PBS. Pictures were captured at intervals of 2 h using a FLUOVIEW FV10i-LIV (OLYMPUS), and then the migration areas were calculated using Image J.

### Preparation of miR-126 incorporated PLGA NP-coated stent

Conventional bare metal stents were coated with chitosan-modified PLGA NPs containing dsRNAs using an electrostatic coating method. dsRNA-incorporating chitosan-modified PLGA NPs were dissolved in distilled water (10% wt/wt), and the stent was immersed in this solution for 10 minutes and dried at low temperature. This procedure was repeated several times to coat a sufficient dose of PLGA NPs onto the stent surface. The amount of RNA per stent is 33.4±0.79 μg for miR-126 and 44.6±1.27 μg for control RNA.

### In vitro release of miR-126 from a miR-126-incorporating PLGA NP-coated stent

miR-126-incorporating PLGA NP-coated stents were immersed in PBS at 37°C. Periodically, PBS was removed and the miR-126 expression levels were measured using qPCR.

### Stent implantation

Male Japanese white rabbits weighing 2.5–3.5 kg were used. Animals were fed a hypercholesterolemic diet at 1 week before intervention and given water ad libitum. All animals received low-dose aspirin for 7 days before intervention and thereafter until sacrifice. Rabbits were anesthetized intramuscularly with a mixture of xylazine and ketamine. After administration of anesthesia, the right carotid artery was surgically exposed, and a 5-Fr introducer sheath (Terumo Co., Tokyo, Japan) was introduced into the artery. A control RNA NP-coated or miR-126 NP-coated stent mounted on a balloon catheter was placed in the iliac artery on the same day (N = 10 vessels/group). The stent delivery system was advanced to the distal portion of the iliac artery using the guide wire, and the stent was deployed to achieve a stent-to-artery-size ratio range of 1.2:1.4. Post-stent deployment angiography was carried out in all animals. At the time of stent implantation, an intravenous bolus of heparin at 100 U/kg was administered to prevent acute thrombosis. Rabbits were then visually monitored for signs of pain, distress, or moribundity (sudden behavioral change, poor posture or ambulation difficulty, loss of hair coat condition, sudden activity change, painful facial expressions, neurological disorders, and cardiopulmonary disorders, according to the Kyoto University Institutional Animal Care and Use Guidelines) and weighed three times a week. Animals reaching these endpoints, or exhibiting at 15% reduction in weight were to be euthanized. However, no rabbit exhibited signs of pain, distress, or moribundity or during the course of evaluation, and all rabbits in this study were humanely euthanized at the fixed time point of 28 days post implantation when follow-up angiography was performed. After administration of anesthesia as before, angiography was performed. Then, the animals were euthanized with a lethal dose of pentobarbital. Stented vessels were harvested and stored overnight at 4°C in buffered formalin.

Optical coherence tomography (OCT) was conducted *ex vivo* on coronary arteries after vessel fixation, using Light lab and a Dragonfly^™^ OCT catheter (St Jude Medical, Stratford-upon-Avon, UK).

The stented vessels were fixed in 4% paraformaldehyde. To prepare cross-sections of stented vessels, fixed arteries were embedded in plastic resin (Technovit 8100, Heraeus Kulzer, Armonk) in accordance with the manufacturer’s instructions and cut into 5 μm sections in each part. Each section was stained with hematoxylin and eosin for histological analysis.

### Statistics

Data are presented as means ± SEM. Statistical significance (P<0.05) was determined between groups using an ANOVA followed by Tukey's Multiple Comparison Test for multiple groups or a Student’s t-test for two groups.

## Results

### Cholesterol conjugated 27-nt dsRNAs to form miR-126 and its delivery using PLGA NPs

**Fig 1:**
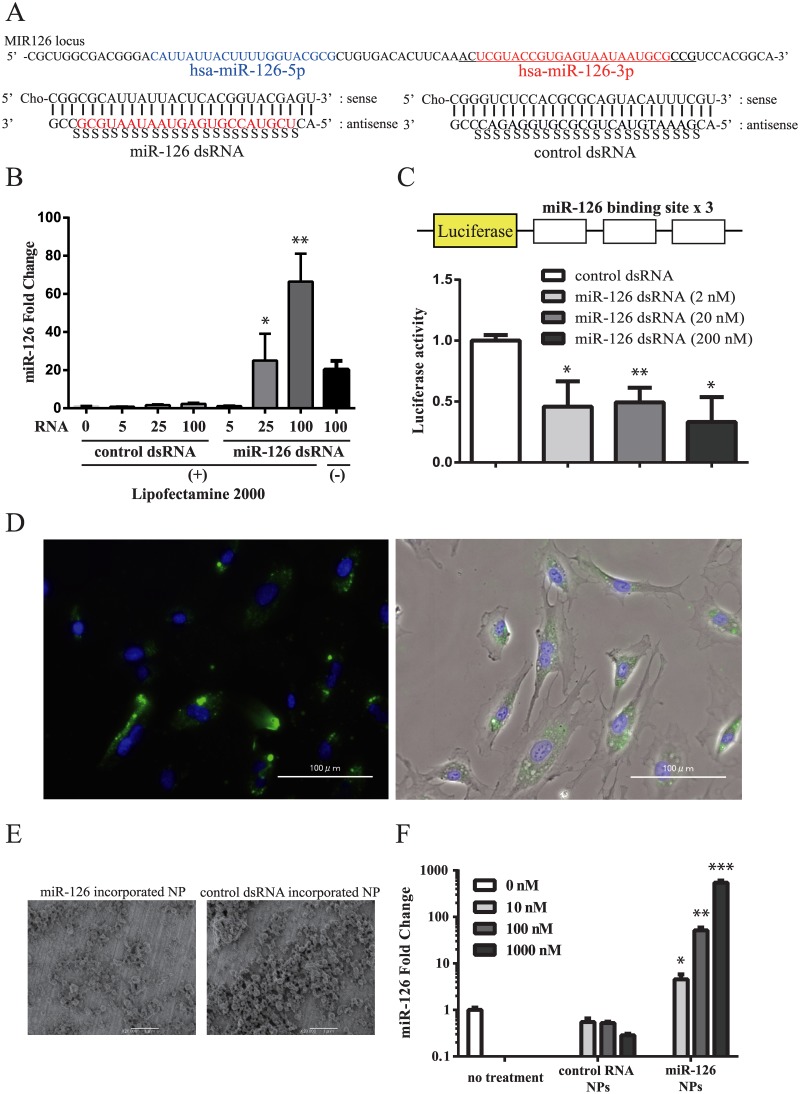
Structure of miR-126 dsRNA and miR-126 expression from miR-126 NPs *in vitro*. (A) Structure of miR-126 dsRNA and control dsRNA. (B) miR-126 expression after the induction of miR-126 dsRNA and control dsRNA using Lipofectamine 2000 in 293T cells. Values are means ± SEM; n = 5 each; *P<0.05, **P<0.01. (C) Luciferase activity of a reporter gene with miR-126 binding sites. Values are means ± SEM; n = 4 each; *P<0.05, **P<0.01. (D) FITC levels in HUVECs after the addition of FITC-NPs. Fluorescence intensity (left panel) and phase contrast image (right panel). (E) Electron micrograph of miR-126 NPs and control NPs. (F) miR-126 expression levels after the addition of miR-126 NPs and control NPs in HUVECs. Values are means ± SEM; n = 4 each; *P<0.05, **P<0.01. ***P<0.001.

The structures of pri-miR-126, miR-126 dsRNA and control scrambled oligonucleotide dsRNA (control dsRNA) are described in the Material and Methods and Fig 1A. We transfected miR-126 dsRNAs and control dsRNAs into 293T cells using Lipofectamine 2000. As shown in Fig 1B, mature miR-126 levels were significantly increased in cells transfected with miR-126 dsRNA compared with control dsRNA. The effect of miR-126 after the induction of these oligonucleotides was measured using the reporter assay. Luciferase genes with three miR-126 binding sites were induced in 293T cells with the transfection of control dsRNA or miR-126 dsRNA. As shown in Fig 1C, luciferase activity was significantly reduced by the induction of miR-126 dsRNA compared with control dsRNA.

We first tried to observe the effect of PLGA NPs to deliver FITC to HUVECs. As shown in Fig 1D, PLGA NPs were efficiently incorporated into HUVECs. Then we produced miR-126 dsRNA and control RNA containing PLGA NPs (miR-126 NPs and control NPs, respectively) (Fig 1E). Addition of miR-126 NPs efficiently induced miR-126 expression in HUVECs compared with control NPs (Fig 1F).

### Increase in growth and migration of HUVECs by miR-126 NPs

**Fig 2:**
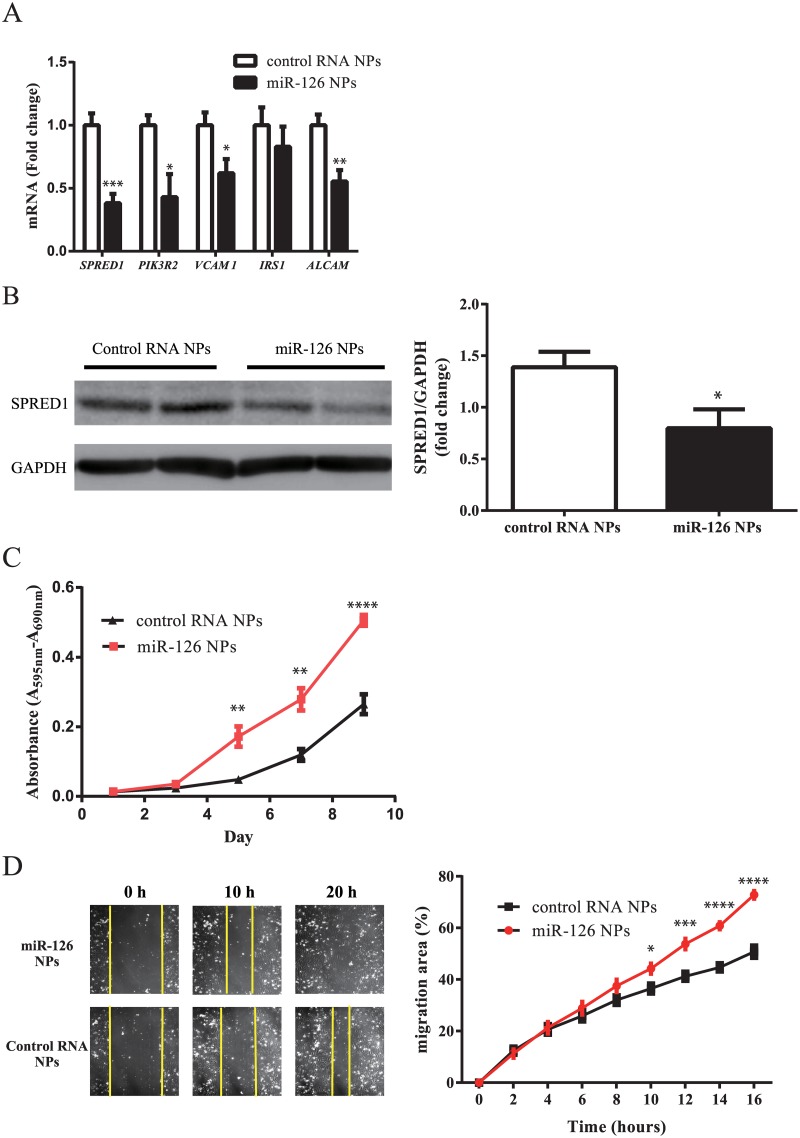
Effect of miR-126 NPs on HUVECs. (A) mRNA expression changes of potential target genes of miR-126 in HUVECs determined by real-time PCR analyses. Values are means ± SEM; n = 5 each; *P<0.05, **P<0.01. ***P<0.001. (B) Protein levels of SPRED1 after addition of control RNA NPs and miR-126 NPs. Values are means ± SEM; n = 6 each; *P<0.05. (C) Proliferation of HUVECs determined by MTT assay. Values are means ± SEM; n = 8 each; *P<0.05, **P<0.01. ****P<0.0001. (D) Photograph of scratch assay and serial changes in the migration area determined using Image J. Values are means ± SEM; n = 8 each; *P<0.05, ***P<0.001. ****P<0.0001.

The effect of miR-126 NPs on endothelial cells was examined in HUVECs. After the induction of miR-126 NPs and control RNA NPs, expression levels of several previously defined miR-126 target genes were measured by RT-PCR. As shown in Fig 2A, the expression levels of SPRED1, PIK3R2, VCAM1, and ALCAM1 were significantly reduced by the induction of miR-126 NPs compared with control RNA NPs. However, IRS1 expression was not changed by miR-126 NPs (Fig 2A). Western blotting analysis indicated that miR-126 NPs significantly reduced the protein levels of SPRED1 (Fig 2B). When cellular proliferation was assessed using an MTT assay, miR-126 NPs significantly increased the proliferation of HUVECs compared with control RNA NPs (Fig 2C). Moreover, the effect on cellular migration activity was examined by the use of FLUOVIEW FV10i-LIV (OLYMPUS). miR-126 NPs significantly increased the migration activity as shown in Fig 2D.

### Decrease in VSMC growth and migration by miR-126 NPs

**Fig 3:**
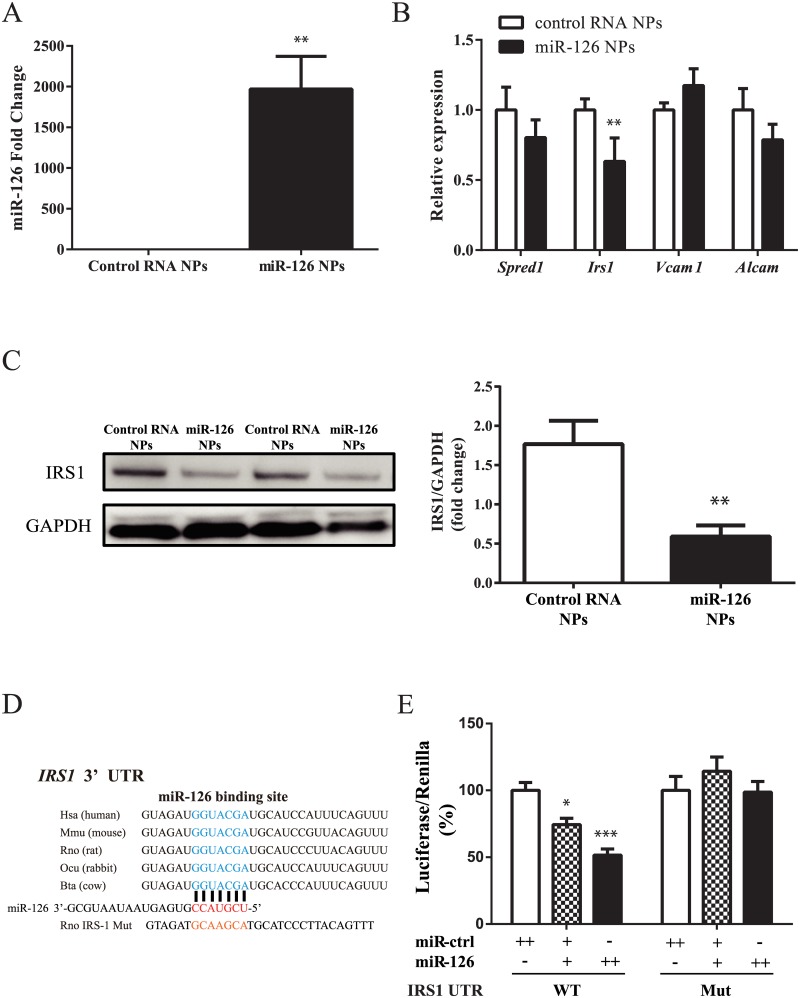
miR-126 NPs target IRS-1 in VSMCs. (A) miR-126 expression levels after the addition of miR-126 NPs and control NPs to VSMCs. Values are means ± SEM; n = 4 each; **P<0.001. (B) mRNA expression changes of potential target genes of miR-126 in VSMCs determined by real-time PCR analyses. Values are means ± SEM; n = 4 each; **P<0.01. (C) Protein levels of IRS-1 after addition of control RNA NPs and miR-126 NPs. Values are means ± SEM; n = 6 each; **P<0.001. (D) Conservation of the miR-126 target site in the 3’-UTR of IRS-1. (E) 3’-UTR reporter assay used to verify the target. Luciferase reporter activity of rabbit IRS-1 gene 3’-UTR constructs with or without mutation of the miR-126 binding site in 293T cells overexpressing miR-control and miR-126; n = 4 each; *p < 0.05 and ***p < 0.001.

**Fig 4:**
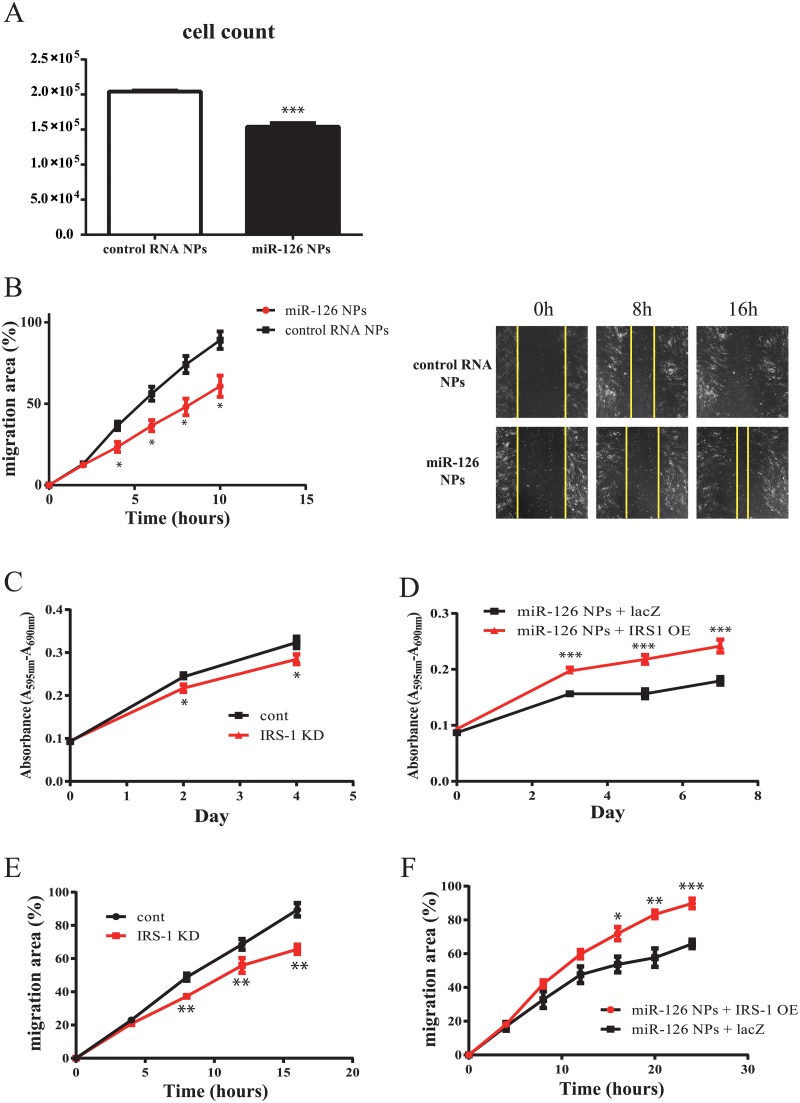
Effect of miR-126 NPs on VSMCs. (A) Proliferation of VSMCs determined by cell count. Values are means ± SEM; n = 6 each; ***P<0.001. (B) Photograph of scratch assay and the serial changes in migration area determined using Image J. Values are means ± SEM; n = 4 each; *P<0.05. (C and D) Proliferation of VSMCs determined using an MTT assay. Values are means ± SEM; n = 6 each; *P<0.05, ***P<0.001. (E and F) Serial changes in migration area determined using Image J. Values are means ± SEM; n = 4 each; *P<0.05, **P<0.01, ***P<0.001.

Next, we analyzed the effect of miR-126 NPs on VSMCs. Primary VSMCs were prepared from rabbit iliac arteries. Because miR-126 is not expressed in VSMCs, miR-126 expression levels were strikingly enhanced by the addition of miR-126 NPs compared with control RNA NPs (Fig 3A). After the incubation with miR-126 NP and control RNA NP, the expression levels of several target genes were measured by RT-PCR. As shown in Fig 3B, the expression level of IRS-1 was significantly reduced by miR-126 NPs compared with control dsRNA NPs. However, SPRED1, VCAM1, and ALCAM expression levels were not changed by miR-126 NPs compared with control dsRNA NPs. We also confirmed that IRS-1 protein levels were reduced by the induction of miR-126 NPs compared with control NPs (Fig 3C). Previously, IRS-1 was reported to be targeted by miR-126 in tumor cells [15]. As shown in Fig 3D, the miR-126 biding site in the 3’-UTR is evolutionally conserved. miR-126 NPs significantly reduced the luciferase activity in cells transfected with the luciferase reporter gene with an IRS-1 3’-UTR. Mutations in the miR-126 binding sites of the IRS-1 3’-UTR abolished the reduction in luciferase activity (Fig 3E). Thus, miR-126 NPs most likely reduced the levels of IRS-1 through its binding to the IRS-1 3’-UTR. Then, the effects of miR-126 on VSMC proliferation and migration was analyzed. As shown in Fig 4A, miR-126 NPs reduced the proliferation of VSMCs compared with control NPs. Migration activity of VSMCs was also reduced by miR-126 NPs compared with control NPs (Fig 4B). Knockdown (KD) of IRS-1 reduced the proliferation of VSMCs, and the effect of miR-126 NPs on VSMC proliferation was reversed by the induction of IRS-1 (Fig 4C and 4D). Migration activity was also reduced by IRS-1 KD, which was abolished by overexpression of IRS-1 (Fig 4E and 4F). Moreover, several genes that indicated VSMCs differentiation were also measured. Taglin and Acta2 were upregulated by miR-126 NPs (S1A Fig), and these levels were enhanced by IRS-1 KD (S1B Fig), accordingly.

### In vivo effect of miR-126 NP-conjugating stents

**Fig 5:**
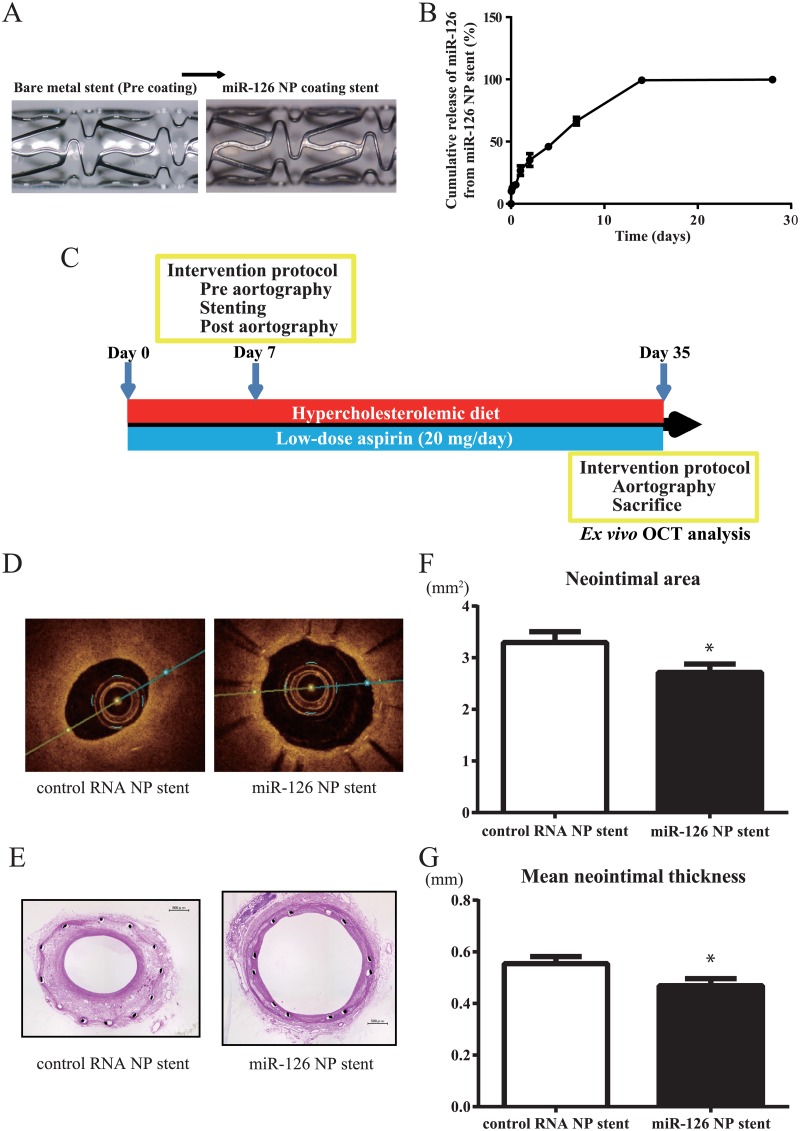
Effect of miR-126 NP-conjugated stents on a rabbit model of neointimal formation. (A) Photograph of an miR-126 NP-conjugated stent. (B) Cumulative release of miR-126 from an miR-126 NP-conjugated stent. (C) Protocol of the *in vivo* rabbit model of neointimal formation. (D) Representative OCT images of a rabbit iliac artery after the implantation of control RNA NP-conjugated stents or miR-126 NP-conjugated stents. (E) Representative images of HE staining of a rabbit iliac artery after the implantation of a control RNA NP-conjugated or miR-126 NP-conjugated stent. (F) Changes in neointimal area of rabbit iliac arteries after the implantation of a control RNA NP-conjugated or miR-126 NP-conjugated stent; n = 8 each; *P<0.05. (G) Changes in mean neointimal thickness of rabbit iliac arteries after the implantation of a control RNA NP-conjugated or miR-126 NPs-conjugated stent; n = 8 each; *P<0.05.

We further evaluated the effect of miR-126 NPs using a rabbit model of neointimal formation. We first conjugated miR-126 NPs and control NPs to bare metal stents as indicated previously (Fig 5A). The cumulative release of miR-126 from a miR-126 NP-conjugated stent is shown in Fig 5B. We delivered these miR-126 NP- and control NP-conjugated stents to a rabbit model of neointimal formation of the iliac artery, in which stenting was performed immediately after endothelial denudation (Fig 5C). Neointimal formation was analyzed by angiography and OCT at 4 weeks after treatment. Vascular stenting with a control NP-conjugated stent was associated with marked neointimal hyperplasia. In contrast, miR-126 NP-conjugated stents inhibited the progression of neointimal hyperplasia (Fig 5D–5G). We measured the levels of miR-126 in rabbit iliac arteries 1 week after the implantation of control NP- and miR-126 NP-conjugated stents. miR-126 levels were higher in samples with miR-126 NP-conjugated stents than control stents. However, there was no statistical difference (S1C Fig).

## Discussion

In these experiments, we designed and constructed a 27-nt dsRNA conjugated with cholesterol, which has high membrane permeability, and this dsRNA formed mature miR-126 after transfection. Moreover, we utilized poly PLGA-NPs for site-specific induction of miR-126. miR-126 NPs significantly reduced the protein expression of SPRED1 in HUVECs and enhanced the proliferation and migration of HUVECs. On the other hand, miR-126 NPs reduced IRS-1 levels in rabbit SMCs and reduced the proliferation and migration of VSMCs via the suppression of IRS-1. Finally, we developed a stent system that eluted miR-126, and this delivery system exhibited significant inhibition of neointimal formation, which indicated the possibility of a novel therapeutic option to prevent restenosis after angioplasty by the induction of miR-126.

For miRNAs whose expression is beneficial for a specific condition, overexpression of such a mature miRNA into the proper tissue could provide a therapeutic benefit by enhancing the regulation of target genes. It was realized that siRNAs resemble a mature miRNA duplex, and in fact, they are functionally interchangeable in terms of RISC action against target mRNAs [16]. Thus, the introduction of synthetic siRNA-like molecules that mimic the Dicer-processed miRNA duplex is a potential method to enhance the expression of desired miRNAs. Therefore, we adopted this method to deliver a 27-nt dsRNA conjugated to cholesterol, which has high membrane permeability, and this dsRNA formed mature miR-126. Moreover, it is known that therapeutic oligonucleotides are generally modified in the phosphate backbone and/or include ribose sugars to increase nuclease resistance and enhance affinity for target RNAs. The phosphorothioate backbone modification replaces a non-bridging oxygen atom with a sulfur atom and extends the half-life of oligonucleotides in plasma from minutes to days [17]. Therefore, we designed a stabilized mature miR-126 through the inclusion of a phosphorothioate backbone modification. We found that transfection of the miR-126 dsRNA significantly enhanced miR-126 expression. For clinical applications of a miR-126 dsRNA, it will be necessary to enhance transfection efficiency and to release oligonucleotides over a period of several weeks. Thus, we incorporated miR-126 dsRNA into PLGA NPs, and the addition of miR-126 NPs into the medium significantly enhanced the expression levels of miR-126 and reduced the expression of target genes.

It is known that miR-126 enhances the proangiogenic actions of VEGF and FGF by repressing the expression of SPRED1, an intracellular inhibitor of angiogenic signaling [11]. We confirmed that miR-126 NPs can repress the protein levels of SPRED1 in HUVECs. Moreover, miR-126 NPs significantly enhanced the proliferation and migration of HUVECs. We also confirmed that IRS-1 is targeted by miR-126 in VSMCs, which mediate the suppression of proliferation and migration of SMCs. This was in agreement with previous reports that IRS1 is translationally inhibited by miR-126 in HEK293 and MCF-7 cells [18]. Overexpression of miR-126 also increased differentiation marker gene expression levels in VSMCs, which was compatible with VSMCs with reduced proliferation. Previously, Zhou et al. indicated that loss of miR-126 reduced the ligation–induced neointimal growth in mice carotid artery [19]. However, their findings are mainly based on the loss-of-function experiment, which are different from our over-expression model.

Another hurdle for miRNA-based therapeutics is the delivery of nucleic acids to the target tissue or cells. Although systemic delivery of siRNAs has already shown promising results [20,21], there is still the possibility of undesirable side-effects. For the local release of miR-126, we conjugated miR-126 NPs to stents, which released mature miR-126 for about 2 weeks locally. We evaluated miR-126 NP-conjugated stents in a rabbit model of neointimal formation. miR-126 NP-conjugated stents significantly inhibited the development of neointimal hyperplasia in rabbits.

There were several limitation of our *in vivo* experiments. We could not detect the changes in potential target gene expression in the stent implanted artery because it was hard to measure endothelial and smooth muscle cell-specific gene expression separately *in vivo*. Moreover, we could not observe the detailed miR-126 release profile in the iliac artery. It is possible that the measurement of miR-126 in the whole iliac artery may ameliorated the expression levels of miR-126.

The present study demonstrates the possible utility of this novel therapeutic option to prevent restenosis and thrombosis after angioplasty.

## Supporting information

S1 Fig mRNA expression changes in VSMC and relative expression of miR-126. (A) mRNA expression changes in VSMC differentiation-associated genes in VSMCs determined using real-time PCR analyses after the addition of miR-126 NPs or control NPs. Values are means ± SEM; n = 4 each; **P<0.01. (B) mRNA expression changes in VSMC differentiation-associated genes in VSMCs determined using real-time PCR analyses with or without knock-down of IRS-1. Values are means ± SEM; n = 4 each; *P<0.05. (C) Relative expression of miR-126 treated in iliac arteries with miR-126 NP-conjugated or control RNA NP-conjugated stents at 1 week after the implantation; n = 3.
(DOCX)
