# Supplementary material for: Prevention of neointimal formation using miRNA-126-containing nanoparticle-conjugated stents in a rabbit model
Source: PLoS One. 2017 Mar 2;12(3):e0172798. doi: 10.1371/journal.pone.0172798 (PMC5333844; doi:10.1371/journal.pone.0172798)
Supplement: S1 Fig — (A) mRNA expression changes in VSMC differentiation-associated genes in VSMCs determined using real-time PCR analyses after the addition of miR-126 NPs or control NPs. Values are means ± SEM; n = 4 each; **P<0.01. (B) mRNA expression changes in VSMC differentiation-associated genes in VSMCs determined using real-time PCR analyses with or without knock-down of IRS-1. Values are means ± SEM; n = 4 each; *P<0.05. (C) Relative expression of miR-126 treated in iliac arteries with miR-126 NP-conjugated or control RNA NP-conjugated stents at 1 week after the implantation; n = 3. (DOCX) [file pone.0172798.s001.docx]

**Supporting Information**

**Prevention of Neointimal Formation Using miRNA-126-Containing Nanoparticle-Conjugated Stents in a Rabbit Model**

**Masayasu Izuhara, Yasuhide Kuwabara, Naritatsu Saito, Erika Yamamoto, Daihiko Hakuno, Yasuhiro Nakashima, Takahiro Horie, Osamu Baba, Masataka Nishiga, Tetsushi Nakao, Tomohiro Nishino, Fumiko Nakazeki, Yuya Ide, Masahiro Kimura, Takeshi Kimura, and Koh Ono**

**S1 Fig. mRNA expression changes in VSMC and relative expression of miR-126.**

(A) mRNA expression changes in VSMC differentiation-associated genes in VSMCs determined using real-time PCR analyses after the addition of miR-126 NPs or control NPs. Values are means ± SEM; n=4 each; **P<0.01. (B) mRNA expression changes in VSMC differentiation-associated genes in VSMCs determined using real-time PCR analyses with or without knock-down of IRS-1. Values are means ± SEM; n=4 each; *P<0.05. (C) Relative expression of miR-126 treated in iliac arteries with miR-126 NP-conjugated or control RNA NP-conjugated stents at 1 week after the implantation; n=3.
